# Continuous Blood Glucose Monitoring Increases Vigorous Physical Activity Levels and Is Associated With Reduced Hypoglycemia Avoidance Behavior In Youth With Type 1 Diabetes

**DOI:** 10.3389/fendo.2021.722123

**Published:** 2021-09-07

**Authors:** Georges Jabbour, Nicola Luigi Bragazzi

**Affiliations:** ^1^Department of Physical Education, College of Education, Qatar University, Doha, Qatar; ^2^Laboratory for Industrial and Applied Mathematics (LIAM), Department of Mathematics and Statistics, York University, Toronto, ON, Canada; ^3^Department of Health Sciences (DISSAL), Postgraduate School of Public Health, University of Genoa, Genoa, Italy

**Keywords:** youth, type 1 diabetes, continuous blood glucose monitoring, insulin pump, vigorous physical activity, child hypoglycemia fear survey behavior, sometimes experience PA hypoglycemia

## Abstract

The primary goal of this study was to explore physical activity (PA) levels, hypoglycemia fear scores and hypoglycemia episodes according to insulin administration and blood glucose monitoring methods in youth with type 1 diabetes (T1D). A self-administered questionnaire was completed by 28 children and 33 adolescents with T1D, and their PA was assessed. Hypoglycemia episodes, fear of hypoglycemia scores, insulin therapy (pump vs. injection) and blood glucose monitoring (continuous blood glucose monitors [CGMs] vs. blood glucose meters) methods are reported in the present work. There were no significant differences in the number of hypoglycemic episodes, child hypoglycemia fear survey behavior or total scores, or any components of the PA profile between youth using injections and those using a pump. However, these variables differed significantly when compared according to blood glucose monitoring method (CGMs vs. blood glucose meters): 41.2 *vs.* 81.8, p<0.01; 1.03 ± 0.05 *vs*. 2.6 ± 0.63, p<0.01; 1.09 ± 0.43 *vs*. 2.94 ± 0.22, p<0.01; and 222 ± 18 *vs.* 49 ± 11, p<0.01 (for total time in vigorous PA in minutes per week), respectively. CGM use correlated significantly with VPA levels (β=0.6; p=0.04). Higher VPA levels were associated with higher child hypoglycemia fear survey behavior scores (β=0.52; p=0.04). The latter correlates negatively with the number of episodes of hypoglycemia in the past 12 months in all category groups. The type of insulin injection was not associated with more activity in youth with T1D. In contrast, CGM use may be associated with increased vigorous PA among T1D youth. Those with higher hypoglycemia fear survey behavior scores engaged in more VPA and had fewer hypoglycemia episodes. Although CGM use ensures continuous monitoring of glycemia during exercise, increasing hypoglycemia avoidance behavior is still a necessary part of exercise management strategies in active youth with T1D.

## Introduction

Type 1 diabetes (T1D), previously termed as juvenile diabetes, represents a complex, multi-factorial, autoimmune disorder, characterized by an impaired production and release of insulin by the beta cells of the islets of Langerhans in the pancreas. Insulin finely tunes glucose uptake and use, contributing to normal blood sugar levels and homeostasis, whereas insulin deficiency results in hyperglycemia (1).

T1D is a global public health concern, generating a dramatic burden, both from a societal and clinical perspective. According to the Global Burden of Disease, from 1990 to 2017, worldwide, the age-standardized incidence and prevalence rates have slightly increased from 5.1 (95% uncertainty interval or UI 4.6–5.6) to 5.4 (95%UI 4.9–6.0), and from 161.7 (95%UI 146.1–180.7) to 164.8 (95%UI 148.4–184.9), respectively. An opposite trend could be observed for the age-standardized rate decreased for mortality and disability-adjusted life year (DALY), which is the computed number of years lost due to ill-health. Both these rates decreased from 5.7 (95%UI 5.2–6.3) to 4.3 (95%UI 4.0–4.7), and from 164.0 (95%UI 151.8–180.7) to 129.4 (95%UI 121.3–137.6), respectively (2).

From an organismal standpoint, T1D is characterized by micro-vascular disease (including diabetic retinopathy and diabetic nephropathy) and macro-vascular damage (such as cardiovascular disease) (3).

Despite the general consensus that physical activity (PA) improves micro- and macro-vascular outcomes in individuals with T1D (4) and helps them achieve their target fitness and glycemic goals (5), motivating this population to engage in regular PA is still a very challenging task (6). One prominent barrier to PA, hypoglycemic episodes, can occur during or after exercise (7) and among active individuals as well as highly trained athletes with T1D (8, 9). Therefore, real-time observation of blood glucose levels and precise adjustments to insulin dosage before, during and following activity constitute a primary recommendation from current research on implementing strategies to engage T1D individuals in safe and effective PA.

Maintaining normal blood glucose levels to avoid and/or reduce hypoglycemia episodes in response to exercise requires tight control of daily insulin injections as well as continuous monitoring of glucose levels. Unfortunately, data regarding hypoglycemia in relation to exercise in T1D individuals are limited to laboratory studies designed to investigate preventative approaches or different exercise regimen (10, 11). Moreover, few studies exist on the potential association of hypoglycemia episodes with PA participation, and even fewer address strategies adopted to prevent and to control blood glucose levels in response to exercise in the real-life context for adults as well as youth with T1D (10–12).

General guidelines to manage exercise in individuals with T1D include regular blood glucose tests, following recommended diets, and applying functional insulin therapy (12). O’Connell et al. (13) and Birkebaek et al. (14) reported significantly lower rates of hypoglycemia episodes over time, which may be explained by modern diabetes management, such as insulin pump therapy and continuous blood glucose monitoring (CGM). Despite these promising technologies in enhancing glycemic control quality/sustainability, it stills unclear whether users will adopt more active lifestyles in response. In youth with T1D, the presence of technology for managing glycemic levels during exercise may constitute a major challenge since appropriate behavioral management is indispensable (15). In addition, other factors, mainly the fear of hypoglycemia, a primary and specific barrier to PA in T1D individuals (16–19) may limit the activities of youth with T1D. The challenge remains to ensure optimal adequacy between the potential advantages of technology and optimal glycemic control to prevent hypoglycemia and to improve PA.

Within this perspective, the present study investigated the association of use of modern diabetes management (insulin therapy method and continuous glucose monitoring) with PA levels in youth with T1D in relation to other PA determinants, mainly the fear of hypoglycemia and reported number of hypoglycemia episodes. We hypothesized that users of modern diabetes management techniques would have i) less fear of hypoglycemia, and ii) increased PA levels than nonusers.

## Methods

### Participants and Study Design

Youth were eligible for participation if they were aged 5-17 years and had been diagnosed with T1D for ≥1 year. The study was conducted at the Pediatric Diabetic Clinic of Dr. Georges-L.-Dumont (Vitalité Health Network), located in Dieppe, Quebec, Canada. Sixty-one children and adolescents with T1D were followed (from a total of ~630 patients registered in all affiliated pediatric clinics of the Vitalité Health Network). The ethics committee of the Vitalité Health Network approved the project. Informed consent documents were reviewed and signed by the parent and/or participant. Of the 61 children and adolescents with T1D recruited, all agreed to take part in the study, representing 100% of the Pediatric Diabetic Clinic of Dr. Georges-L.-Dumont**’**s clientele. All completed a self-administered questionnaire that was distributed by kinesiology students from the University of Moncton in-person at the clinic.

Age and sex were obtained using a self-report questionnaire completed by the child/adolescent. A nurse evaluated height and weight on site. Age- and sex-specific body mass index (BMI) percentiles were calculated according to the US Centers for Disease Control and Prevention growth charts (20, 21). The number of years since the patient’s diabetes diagnosis was also calculated, and mean HbA1c values over the preceding 3 months were preliminarily recorded. The method of insulin administration (injection vs. pump) was reported, and the patients answered “yes” or “no” to whether they used CGM or simple self-monitoring using a gluco meter.

Participants completed the Children’s Hypoglycemia Fear Survey (CHFS) (22), which consists of two components: the Worry Subscale (CHFS-W) (15 items) and the Behavior Subscale (CHFS-B) (10 items). Each item is scored from 0 to 4; 0 represents the least fear of hypoglycemia and 4 the most. An average per-item score is generated and results in behavior and worry subscale mean scores and a total mean score. Moreover, the frequency of hypoglycemia survey (23) was used to collect retrospective data on participants’ experience of hypoglycemia over the past 12 months.

To obtain the activity profile, we used the questionnaire from Cycle 2 of the Canadian Health Measures Survey (24). This questionnaire was developed and administered in two versions: one for children under 12 and another for adolescents 12 years and older. In this questionnaire, children and adolescents reported how many hours per day they usually spend engaged in sedentary activities, such as using a computer, playing video games, or watching TV/videos. For the data analysis, the three categories “none”, “<1 h/day”, and “1-2 h/day” were recoded as “≤2 h/day”, and the other categories (“3-4 h/day”, “5-6 h/day”, and “≥7 h/day”) were recoded as “>2 h/day”, which is the closest possible threshold within these categories to the cutoff, according to the Canadian guidelines for screen time (<2 h/day) (25).

Next, the average minutes per day spent in various forms of PA were derived. Using the World Health Organization norms on metabolic equivalents of task (METs) (26) and Ainsworth’s Compendium of Physical Activities for children (27), activities were categorized as low (≤3 METs), moderate (3< METs ≤6), or vigorous (>6 METs) in intensity.

### Statistical Analysis

The analyses were performed using SPSS v. 21 software (IBM, Armonk, New York, USA). The data are presented as the means (standard deviations). Normality was tested using the Kolmogorov–Smirnov test. Independent samples t-tests were used to assess differences in mean outcome scores within and among group categories. We used multiple linear regression to model the mean outcomes for each exposure of interest. For both linear and logistic regressions, the independent variables considered in the regression models were total time in moderate to vigorous PA (MVPA) per week in minutes and total time in vigorous PA (VPA) per week in minutes. Pearson correlations were used to assess the association between the number of hypoglycemia episodes and hypoglycemia fear behavior subscale score. A value of p<0.05 was set as the level of statistical significance.

## Results

The characteristics of the study participants are presented in **Table 1**. Among the children, 68% of subjects using insulin pumps were boys, and 42% of subjects using CGM were boys. Anthropometric variables (height, weight, BMI percentile) are displayed in **Table 1**. There were significant differences in weight, BMI and glucose checks per day between insulin pump vs. insulin injection methods and between CGM vs. blood glucose meters (**Table 1**). The percentage of self-reported frequency of hypoglycemia episodes in the past 12 months is presented in **Table 1**.

**Table 1:** Participant characteristics.

	Insulin treatment method			Blood glucose monitoring		
	Insulin injections(n=39)	Insulin pump (n=22)	p-value	CGM (n=14)	Blood glucose meters (n=47)	p-value
**Sex *(% boys)***	48	68 ^a^	*<0.01*	42 ^a c^	57	*<0.01*
**Age *(years)***	11.9 ± 1.8	12.4 ± 2.2	*0.31*	13.8 ± 3.1	14.6 ± 1.2	*0.54*
**Weight *(kg)***	48.6 ± 4.6	52.1 ± 1.8 ^a^	*<0.01*	55.1 ± 3.3 ^a^	58.6 ± 4.8	*<0.01*
**Height *(cm)***	151 ± 9.1	158 ± 6.1	*0.39*	162 ± 4	168 ± 9	*0.48*
**BMI (*percentile*)**	88 ± 04	85 ± 0.7	*0.12*	75 ± 08 ^a^	64 ± 04	*<0.01*
**HbA1c *(%)***	7.7 ± 3.1	6.8 ± 1.4 ^a^	*<0.01*	7.3 ± 2.2	7.8 ± 1.4	*0.29*
**Glucose checks/day**	6.4 ± 3.9	4.6 ± 2.1 ^a^	*<0.01*	2.6 ± 1.1 ^a b c^	7.1 ± 4.1	*<0.01*
**Number of episodes of hypoglycemia in past 12 months**	20.2 ± 6.4	15.8 ± 4.1 ^a^	*<0.01*	18.7 ± 2.7	19.1 ± 4.6	*0.73*
**Self-reported frequency of hypoglycemia (%) in past 12 months**						
*Never*	11	9	*0.49*	39 ^a b c^	11	*<0.01*
*Less than once/year*	3	6 ^a^	*<0.05*	9	10	*0.33*
*1-3 times/year*	22	16 ^a^	*<0.05*	8	19	*0.48*
*4-12 times/year*	21	11 ^a^	*<0.01*	3 ^a b c^	16	*<0.01*
*More than once/month*	38	24 ^a^	*<0.01*	4 ^a b c^	18 ^b c^	*<0.01*
*More than once/week*	25	14 ^a^	*<0.01*	37 ^a b c^	26 ^c^	*<0.01*
**Sometimes experience PA hypoglycemia (%)**	78.9	80.5	*0.51*	41.2 ^a b c^	81.5	*<0.01*
**Sometimes experience PA hyperglycemia (%)**	31.6	56.4 ^a^	*<0.01*	47.6 ^a b c^	58.8 ^b^	*<0.01*
**Hypoglycemia fear survey scores**						
*Child hypoglycemia fear survey-behavior*	2.08 ± 0.35	2.1 ± 0.76	*0.34*	1.03 ± 0.05 ^a b c^	2.6 ± 0.63	*<0.01*
*Child hypoglycemia fear survey-worry*	1.98 ± 0.41	2.01 ± 0.33	*0.42*	2.01 ± 0.11	2.46 ± 0.53	*0.33*
*Child hypoglycemia fear survey-total*	1.88 ± 0.31	1.94 ± 0.41	*0.29*	1.09 ± 0.43 ^a b c^	2.94 ± 0.22 ^b c^	*<0.01*
**Physical activity and sedentary time**						
*Total screen time·day^−1^ (h) (TV, video games, computer)*	2.81 ± 1.4	2.76 ± 1.8	*0.64*	3.01 ± 1.1	3.3 ± 1.1	*0.41*
*Total time in MVPA per week^-1^ (min)*	364 ± 77	377 ± 104	*0.37*	346 ± 107	333 ± 98	*0.33*
*Total time in VPA per week^-1^ (min)*	136 ± 48	141 ± 31	*0.48*	222 ± 18 ^a b c^	49 ± 11 ^b c^	*<0.01*
*Number of MVPA bouts per week (min)*	2.9 ± 2.1	3.1 ± 0.9	*0.32*	4.1 ± 3.2	3.9 ± 1.9	*0.27*
*Number of VPA bouts per week (min)*	1.9 ± 1.2	2.1 ± 0.9	*0.39*	3.7 ± 1.9 ^a b c^	1.1 ± 0.1 ^b c^	*<0.01*

Values are means (standard deviation). CBGM, continuous blood glucose monitoring; BMI, body mass index; HbA1c, glycated hemoglobin; PA, physical activity; n[%], number, percentage; MVPA, moderate to vigorous physical activity; VPA, vigorous physical activity; h, hour; min, minute.

Significant difference between groups for each category (a: P < 0.01); Significant difference with insulin injection group (b: P < 0.01); Significant difference with insulin pump group (c: P < 0.01).

Self-reports of the frequency of hypoglycemia episodes were explored further by insulin regimen group and by blood glucose monitoring methods. The data in **Table 1** show that the majority of youth had experienced one or more episodes of hypoglycemia regardless of the grouping. However, youth in the insulin pump group reported fewer hypoglycemia episodes (i.e., “1-3 times/year” and “4-12 times/year”) than those who used injections (who more frequently reported having hypoglycemia “more than once/week” and high “more than once/month”) (**Table 1**). However, youth reported fewer episodes of hypoglycemia in all categories when using CGM (**Table 1**) with only one exception in the “more than once/week” category (37% vs 26%). In the CGM group, 41.2% of youth reported sometimes experiencing hypoglycemia during PA, while this figure was 78.9%, 80.5% and 81.5% in insulin injection, insulin pump and blood glucose meters groups, respectively (**Table 1**). The number of episodes of hypoglycemia in the past 12 months is reported in **Table 1**. Participants using insulin pumps reported fewer episodes than insulin injection users (15.8 ± 4.1 vs. 20.2 ± 6.4, p<0.01). For the blood glucose monitoring category, no differences were reported between the CGM and blood glucose meter groups (**Table 1**).

The CHFS-B and CHFS total scores were significantly lower in the CGM group than in all other groups (**Table 1**). For CHFS total scores, the blood glucose meter group had the highest value compared with the CGM (p<0.01), insulin pump (p<0.01) and insulin injection categories (p<0.01) (**Table 1**). Regardless of the insulin administration methods, youth with T1D had similar PA levels and sedentary habits (**Table 1**). However, youth using CGM spent more time in VPA (p<0.01) and had more VPA bouts per week (p<0.01) than youth in all other categories did (**Table 1**).

**Figure 1:**
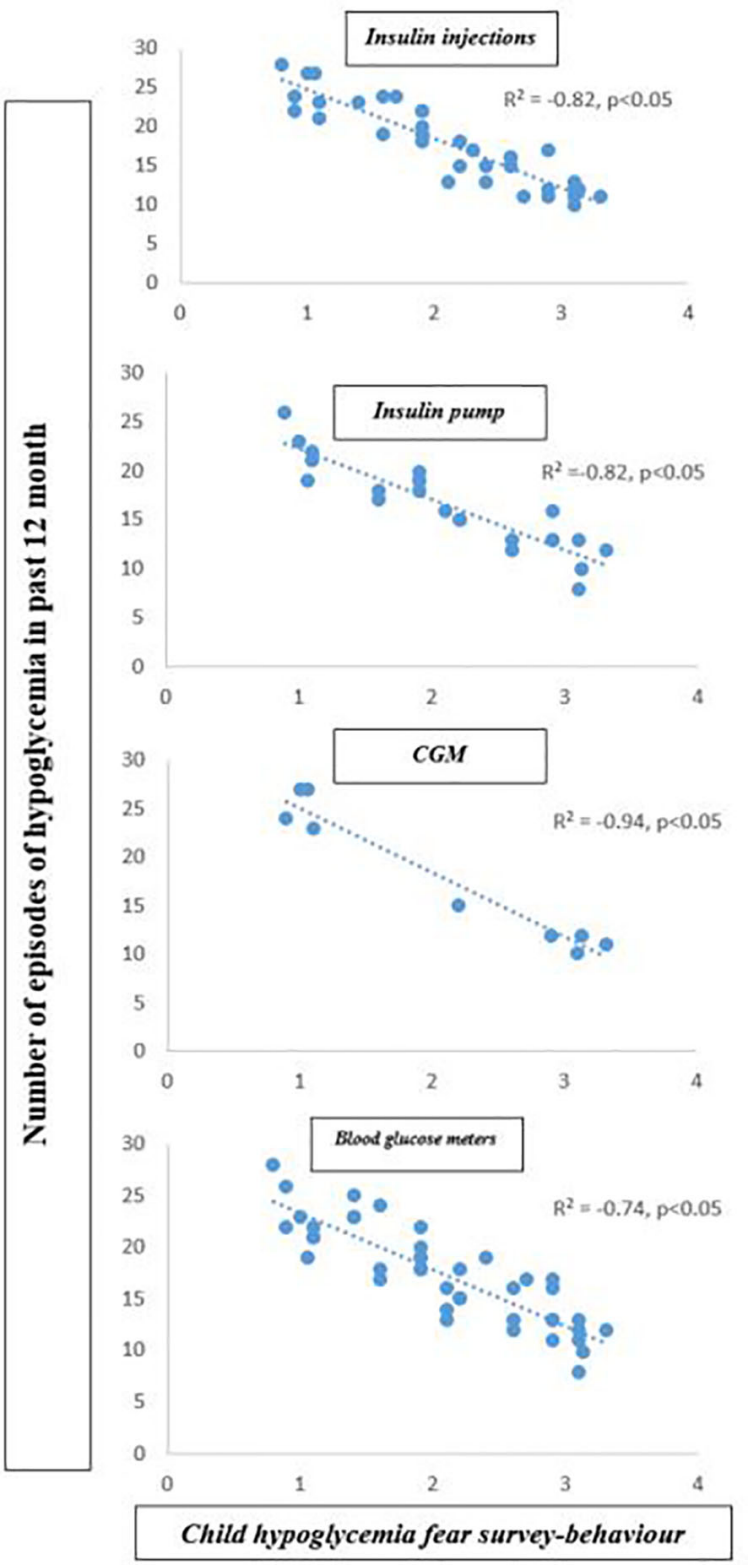
Correlation between the number of hypoglycemia episodes and child hypoglycemia fear survey behavior subscale score.

Our linear regression analysis (**Table 2**) showed that participants using CGM spent significantly more time in VPA per week than participants using blood glucose meters (β= 0.36, p=0.04). Moreover, use of a blood glucose meter was negatively associated with VPA (β= -0.32, p=0.05). A higher CHFS-B score and a higher CHFS total score were associated with high VPA (β= 0.52, p=0.04 and β= 0.43, p=0.08). Finally, higher CHFS-B scores were associated significantly with hypoglycemia episode numbers in all category groups (R2=-0.82 for insulin injection; R2=-0.82 for insulin pump; R2=-0.94 for CGM and R2=-0.74 for blood glucose meters; p<0.05) (**Figure 1**).

**Table 2:** Standardized regression summary for physical activity time per week with other relevant variables.

	Physical activity in week^-1^ (min)	
	Total time in MVPA/week^-1^ (min)	Total time in VPA/week^-1^ (min)
**Insulin injection use**	R^2^ adj. = 0.56; β= 0.04; p=0.68	R^2^ adj. = 0.71; β= 0.06; p=0.66
**Insulin pump use**	R^2^ adj. = 0.67; β= 0.04; p=0.59	R^2^ adj. = 0.73; β= 0.05; p=0.88
**CGM use**	R^2^ adj. = 0.56; β= 0.06; p=0.77	R^2^ adj. = 0.043; β= 0.36; p=0.04*
**Blood glucose meters**	**R^2^ adj. = 0.42; β= 0.44; p=0.02**	R^2^ adj. = 0.061; β= -0.32; p=0.05*
**Sometimes experience PA hypoglycemia (%)**	R^2^ adj. = 0.072; β= -0.31; p=0.02*	R^2^ adj. = 0.79; β= 0.05; p=0.68
**Child hypoglycemia fear survey-behavior subscale score**	R^2^ adj. = 0.64; β= 0.04; p=0.55	R^2^ adj. = 0.049; β= 0.52; p=0.04*
**Child hypoglycemia fear survey-worry subscale score**	R^2^ adj. = 0.44; β= 0.06; p=0.53	R^2^ adj. = 0.59; β= 0.07; p=0.64
**Child hypoglycemia fear survey-total score**	R^2^ adj. = 0.76; β= 0.03; p=0.33	R^2^ adj. = 0.081; β= 0.43; p=0.08*

*denotes significant relationship.

## Discussion

To the best of our knowledge, this study is the first to examine hypoglycemia episodes, fear of hypoglycemia and PA levels and their association with insulin administration and blood glucose monitoring methods in youth with T1D. While insulin administration methods, e.g., pump vs. injection, did not seem to affect PA levels in youth with T1D, CGM users engaged in more VPA and had lower individual CHFS-B scores than other groups. Our study indicated that CHFS-B scores were negatively associated with the number of episodes of hypoglycemia. Thus, CGM users who are more engaged in VPA experience less fear of hypoglycemia as a subjective barrier to exercise despite a high number of episodes of hypoglycemia in the past 12 months. Because CGM allows continuous glucose monitoring, CGM users may have adopted a lower degree of hypoglycemia avoidance behavior and were therefore more likely to experience exercise-induced hypoglycemia. Further study is required to confirm this hypothesis.

With extensive use of modern diabetes technology, especially for those managing T1D with pump therapy and/or continuous glucose monitors, PA practices among individuals with T1D have become better supported, especially in regard to hypoglycemia prevention and management (28). Scientific advancements resulting in the development of sophisticated statistical techniques, based, for example, on machine learning algorithms (including neural networks), have enabled to forecast/nowcast changes in blood glucose levels associated with PA, making it easier to control exercise-induced hypoglycemia (29). Moreover, serious games, like video games and other interactive technologies, have been devised specifically to support youth with T1D, empowering them and promoting positive behavioral changes, enhancing health-related literacy, and improving chronic disease self-management, as well as increasing compliance with the treatment. A qualitative study conducted in a Brazilian sample of children aged 7-12 years confirmed the effectiveness of mini-games in achieving adequate T1D-related knowledge levels and adopting appropriate lifestyles and behaviors (30). The unique combination and convergence of precision medicine and self-centered approaches, together with significant progress in the knowledge of exercise and T1D, with the development of guidelines for maximizing training protocols, and optimizing post-exercise recovery and adaption (31) have substantially improved the quality of life of T1D patients.

Our study reveals that there were no significant differences in PA levels between youth with T1D using injections or a pump. Similar results have been reported previously in studies of the association between insulin administration methods and PA levels in individuals with T1D (19, 32). Such results allow us to suggest that despite the advantage of using insulin pumps, especially regarding blood glucose control, youth benefiting from this type of treatment do not engage in more MVPA or VPA.

Interestingly, while the insulin treatment method was not associated with PA levels, we observed that youth who were using CGM had higher VPA levels. For Galderisi et al. (33), the use of CGM, as it mitigates the fear of exercise-induced hypoglycemia through constant observation of blood glucose, allows children to feel safer during PA and therefore may help in increasing PA among this population. To the best of our knowledge, no study has explored the impact of CGM use on PA levels in a real-life setting. However, a review by Riddell and Perkins (34) showed that the use of real-time CGM might be particularly useful in modifying a patient’s PA habits, thereby ultimately contributing to improvement in glycemic control. Alcántara-Aragón (35) noted that CGM offers opportunities to improve T1D individuals’ self-management, allow observation of the trends in their glycemic control, and prevent hypoglycemia. This latter is still the most important barrier for children and adolescents to engage in PA (17, 18, 36).

Our results revealed that youth had fewer episodes of hypoglycemia in all categories when using CGM, and 41% reported that they “sometimes experienced PA hypoglycemia.” These promising trends were compromised by the fact that 37% of participants using CGM experienced hypoglycemia more than once per week. In parallel, we also reported in all category groups that youth with T1D who displayed higher hypoglycemia avoidance behavior had fewer episodes of hypoglycemia in the past 12 months (**Figure 1**) and vice versa. Interestingly, CGM users had a lower CHFS-B score than other groups and a mean range of numbers of episodes of hypoglycemia in the past 12 months of approximately 18.7 ± 2.7. In addition, a significant positive association for CGM use with total time in VPA per week (R^2^ adj. = 0.043; β= 0.36; p=0.04) and for CHFS-B score with total time in VPA per week (R^2^ adj. = 0.049; β= 0.52; p=0.04) were observed in this study. A recent study by (37) demonstrated that in a generally active cohort of youth with T1D, increased hypoglycemia avoidance behavior was associated with higher PA levels, suggesting that those with more fear of hypoglycemia intervene more to specifically avoid exercise-associated hypoglycemia. The above results highlight the fact that CGM appears to hold promise as a motivational tool to improve physical activity in youth with T1D. In contrast, a lack of hypoglycemia avoidance behavior, frequently observed among CGM users, might be detrimental to glucose monitoring and lead to increased hypoglycemia episodes. Although the fear of hypoglycemia was the most commonly reported barrier to PA in T1D individuals, as noted in previous studies, behavior change interventions are required to optimize CGM efficacy.

Important limitations to this study include the small sample size, especially for those using CGM devices. The next planned tests will be carried out on a larger population using a follow-up design. A valuable supplement to the present research would be the use of an intervention based on an educational approach with participants, and considering other confounding factors such the device label, participants’ gender, social support and educational background.

In summary, this observational study conducted in a “real-life” setting revealed higher VPA levels in CGM users than in other groups. However, though CGM confers an “automatic” continuous glucose measurement for youth with T1D, it does not prevent the occurrence of hypoglycemia among users. Therefore, more hypoglycemia avoidant behavior is likely required to prevent the adverse effects of exercise practices on glycemic control.

## Data Availability Statement

The raw data supporting the conclusions of this article will be made available by the authors, without undue reservation.

## Ethics Statement

The studies involving human participants were reviewed and approved by The ethics committee of the Vitalité Health Network. Written informed consent to participate in this study was provided by the participants’ legal guardian/next of kin.

## Author Contributions

GJ contributed to the conception and design of the study and to the data collection. GJ and NB performed data analysis and interpretation. GJ drafted the manuscript. All authors contributed to the article and approved the submitted version.

## Conflict of Interest

The authors declare that the research was conducted in the absence of any commercial or financial relationships that could be construed as a potential conflict of interest.

## Publisher’s Note

All claims expressed in this article are solely those of the authors and do not necessarily represent those of their affiliated organizations, or those of the publisher, the editors and the reviewers. Any product that may be evaluated in this article, or claim that may be made by its manufacturer, is not guaranteed or endorsed by the publisher.
